# PPARs, RXRs, and Stem Cells

**DOI:** 10.1155/2007/93578

**Published:** 2007-12-31

**Authors:** Z. Elizabeth Floyd, Jeffrey M. Gimble

**Affiliations:** Pennington Biomedical Research Center, Louisiana State University System, Baton Rouge, LA 70808, USA

Welcome to this special issue of *PPAR Research: PPARs, RXRs, and Stem Cells*. Within the past decade, there has been a burgeoning interest regarding the mechanisms regulating stem cell regeneration and differentiation in embryonic and adult tissues. Recent studies have identified stem or progenitor cells within most, if not all, somatic tissues. Cell biologists have explored a number of transcriptional regulatory pathways in the context of stem cell self-renewal and lineage commitment. While there has been a wealth of attention given to the Wnt pathway, Oct4, nanog, and STAT transcription factors in this context, the role of PPARs and related nuclear hormone receptors in regulating stem cells remains relatively unexplored. The current issue of *PPAR Research* has called for manuscripts that will spotlight the PPAR-Stem Cell relationship. We are fortunate to have received a mixture of excellent primary research manuscripts and comprehensive review articles from experts in the field. Mullen, Gu, and Cooney (Houston, Tex) have explored the role of nuclear hormone receptors in murine embryonic stem cell differentiation and function. Purton (Boston, Mass) has comprehensively reviewed the literature concerning the role of retinoid receptors in hematopoietic stem cells. Casteilla, Cousin, and Carmona (Toulouse, Fla) evaluate the classical role of PPARγ as an adipogenic regulator in adipose tissue-derived stromal/stem cells (ASCs). Three investigators use bone marrow-derived mesenchymal stem cell (MSC) models. Isales et al. (Augusta, Ga) provide novel findings relating to the role of mystatin and GILZ on adipogenesis in response to PPAR ligands. Shockley et al. (Bar Harbor, Me & Little Rock, Ark) report the transcriptomic response of MSCs to PPARγ agonists. Duque, Rivas, and Akter (Montreal, Canada) describe a role for farnesylation in modulating MSC adipogenesis. Finally, Cimini et al. (L’Aquila, IT) provide novel insights into the effect of PPARγ during neural stem cell (NSC) astroglial differentiation. We hope that this issue will stimulate other investigators to pursue novel avenues related to the converging themes of PPARs, nuclear hormone receptors, and stem cell biology. The outcomes of such investigations will have far reaching implications regarding fundamental questions relating to normal development, tumor biology, and tissue engineering and regenerative medicine.

*Z. Elizabeth Floyd* *Z. Elizabeth Floyd* *Jeffrey M. Gimble* *Jeffrey M. Gimble*

